# A Pilot Study on the Evaluation of Hetafu Mouthwash in Oral Biofilm of Orthodontic Patients: A Clinical Trial

**DOI:** 10.7759/cureus.107880

**Published:** 2026-04-28

**Authors:** V Vijaykumar, Sathi Rami Reddy Mora, Santha Kumari, Thulasidasan Arumugam, Kuldeep DMello, Mohamed Rahmankhan N

**Affiliations:** 1 Department of Orthodontics and Dentofacial Orthopedics, Adhiparasakthi Dental College and Hospital, Melmaruvathur, IND

**Keywords:** cariogenic bacteria, commensal bacteria, hetafu mouthwash, oral biofilm, orthodontic patients

## Abstract

Introduction

Orthodontic patients are at a high risk of plaque buildup and alterations in oral microbiota due to appliance‑related retention sites. Antimicrobial mouth rinses containing bioactive components have been proposed as adjuncts to support oral microbial balance during treatment. This pilot study evaluated the effect of Hetafu mouthwash on selected beneficial and cariogenic bacteria in patients undergoing fixed orthodontic therapy.

Methods

A prospective clinical trial was conducted on patients undergoing orthodontic treatment at Adhiparasakthi Dental College and Hospital who met the inclusion criteria and provided informed consent. Ethical approval was obtained from the Institutional Ethical Committee of Adhiparasakthi Dental College and Hospital, Melmaruvathur (ethical approval number: ECR/1742/APDCH/ORTHO/FM 05/TN2025). The study included a total of 20 patients. The participants were instructed to use 10 mL of Hetafu mouthwash, rinsing for 30 seconds, two times daily for 240 days without altering their routine oral hygiene practices. Plaque samples were collected at the start of the study, at 60 days, and at 240 days. The samples were cultured on selective media to quantify four target organisms: *Streptococcus mutans*, *Streptococcus oralis* (cariogenic bacteria), *Lactobacillus sporogenes*, and *Streptococcus salivarius* (beneficial commensals). Colony‑forming units were recorded at each time point, and the data were subjected to statistical analysis.

Results

Statistical analysis was conducted using SPSS software version 26.0 (IBM Corp., Armonk, NY), with changes in bacterial counts over time analyzed through the Friedman test, followed by post hoc pairwise comparisons performed using Dunn’s test with Bonferroni modification, and significance set at p < 0.05. A significant and progressive reduction was observed in *Streptococcus mutans* counts across all three time points (p = 0.001), with each interval showing a statistically meaningful decline. *Streptococcus oralis* also demonstrated a marked decrease over time (p = 0.001), with significant differences between all follow‑ups. In contrast, *Streptococcus salivarius* exhibited a steady and significant increase, with the highest levels recorded at 240 days (p = 0.001). *Lactobacillus sporogenes* showed a modest rise, reaching statistical significance only between baseline and the final follow‑up (p = 0.001).

Conclusion

The use of Hetafu mouthwash for 240 days resulted in a significant reduction in *Streptococcus mutans* and a marked increase in *Streptococcus salivarius*, reflecting a shift toward a more favorable oral microbial profile in orthodontic patients. These preliminary findings indicate that the Hetafu mouthwash may serve as a simple adjunct to support oral hygiene during fixed appliance therapy. Future studies with larger sample sizes are necessary to confirm these findings.

## Introduction

Fixed orthodontic appliances often create a complex environment that favors the accumulation and maturation of oral biofilm. Brackets, bands, and arch wires increase plaque retention and hinder effective mechanical cleaning, leading to a shift in microbial composition toward cariogenic and periodontopathogenic species [1]. This is strongly associated with enamel demineralization, white spot lesions, gingival inflammation, and increased caries risk during orthodontic treatment. Orthodontic patients therefore require enhanced preventive strategies beyond conventional mechanical plaque control to maintain oral health throughout treatment [2].

Recent research has explored the use of oral supplements as adjunctive tools to modulate the oral microbiome. Many of these supplements have demonstrated the ability to reduce overall bacterial load or suppress specific pathogenic species [3]. However, most existing studies focus primarily on decreasing harmful bacteria, with limited attention to how such interventions influence the growth dynamics of beneficial commensal organisms that contribute to oral ecological stability [4].

Hetafu mouthwash represents a newer category of pH‑neutral, edible probiotic rinses formulated to support oral health without the irritation associated with conventional alcohol‑based mouthwashes. Its combination of probiotics, prebiotics, essential oils, and enamel‑supporting minerals is designed to support oral health by influencing the overall makeup of the microbial community rather than simply lowering bacterial numbers [5]. Preliminary investigations on Hetafu formulations have reported a decrease in cariogenic microbial levels, accompanied by improvements in salivary characteristics and gingival health [6].

In spite of these findings, no study has specifically evaluated how Hetafu mouthwash affects both beneficial and harmful bacteria in orthodontic patients, a population uniquely susceptible to biofilm‑related complications. Beneficial commensals such as *Streptococcus salivarius* and *Lactobacillus sporogenes* play important roles in maintaining oral microbial homeostasis, competing with pathogens, and supporting mucosal health [7]. Conversely, pathogenic species such as *Streptococcus mutans* and *Streptococcus oralis* are key contributors to enamel demineralization and caries progression [8].

Despite increasing interest in pH‑neutral, probiotic‑based mouth rinses for maintaining oral health, no clinical trial has specifically examined Hetafu mouthwash and its role in the oral biofilm composition of orthodontic patients.

Therefore, this pilot study is designed to evaluate the efficacy of Hetafu mouthwash in modulating the growth of beneficial oral bacteria (*Streptococcus salivarius* and *Lactobacillus sporogenes*) and harmful bacteria (*Streptococcus mutans* and *Streptococcus oralis*) in orthodontic patients. The objective of this study was to evaluate the effect of Hetafu mouthwash on oral microbial flora in orthodontic patients. The outcome measures included the assessment of changes in cariogenic and beneficial bacterial counts over time at different intervals. The results may give preliminary data supporting the use of Hetafu mouthwash as an adjunctive oral hygiene aid for individuals undergoing orthodontic treatment.

## Materials and methods

This study, designed as a prospective pilot clinical study, was conducted in the Department of Orthodontics and Dentofacial Orthopedics at Adhiparasakthi Dental College and Hospital, Melmaruvathur, over a period of eight months from May 2025 to January 2026. Ethical approval was obtained from the Institutional Ethical Committee of Adhiparasakthi Dental College and Hospital, Melmaruvathur (ethical approval number: ECR/1742/APDCH/ORTHO/FM 05/TN2025), and the study was conducted after obtaining consent from the parents/guardians of the patients included in the study. This clinical trial was registered with the Clinical Trials Registry of India (REF/2026/03/126254).

Since the present study was designed as a pilot study due to the differential composition of the mouthwash, a formal sample size calculation was not performed. A sample size of 20 participants was considered appropriate based on recommendations for pilot studies, which suggest that a minimum of 12 subjects per group is sufficient to estimate variability and assess feasibility [9]. The sample consisted of patients undergoing fixed orthodontic treatment at Adhiparasakthi Dental College and Hospital.

The inclusion criteria were patients with full permanent dentition, excluding third molars, having good periodontal health and currently undergoing fixed appliance orthodontic therapy with good to fair oral and general health [5]. The study excluded individuals presenting with noticeable facial deformities, severe malocclusion, or any history of periodontal breakdown. Patients with parafunctional habits, a previous history of maxillofacial surgery or jaw trauma, or known allergies to any of the mouthwash ingredients were also excluded [5].

Hetafu’s CUTE Mouthwash (edible probiotic mouthwash) is a unique blend of probiotics (*Bacillus coagulans*), which actively combat harmful microorganisms through competitive inhibition, antimicrobial production, and immune modulation; prebiotics (fructose oligosaccharide, FOS), which serve as substrates for beneficial bacteria, fostering a microbial environment that supports oral and systemic health; and essential oils and enamel-supporting minerals [5]. It comes in both powder and liquid form. Hetafu is a Department for Promotion of Industry and Internal Trade (DPIIT)-recognized startup, which is Food Safety and Standards Authority of India (FSSAI)-certified, International Organization for Standardization (ISO)-certified, Indian Dental Association (IDA)-accepted, and Food and Drug Administration (FDA)-approved. The Hetafu manufacturers (operating under Lasarkaali Life Sciences Pvt. Ltd.) claim the mouthwash to be edible (safe to swallow); pH-neutral (7.62); chemical-free; clinically designed to protect delicate mouths without burning, staining, or disrupting good bacteria; and gentle enough for kids. Thus, Hetafu mouthwash is specifically engineered to promote oral health.

All enrolled patients were instructed to use 10 mL of Hetafu mouthwash, rinsing for 30 seconds two times daily for a total duration of 240 days, and patients were instructed not to change their routine oral hygiene practices (toothbrushing method, frequency, and toothpaste brand) during the study period [10].

Oral plaque samples were collected from each participant at three time points: baseline, first day (prior to the initiation of Hetafu mouthwash), 60th day, and 240th day. Plaque samples were obtained from standardized sites around the brackets of maxillary and mandibular teeth using sterile cotton swabs [5]. These samples were immediately transferred into sterile tubes containing phosphate‑buffered saline (PBS) solution to facilitate dilution and transported to the microbiology laboratory for culture.

**Figure 1:**
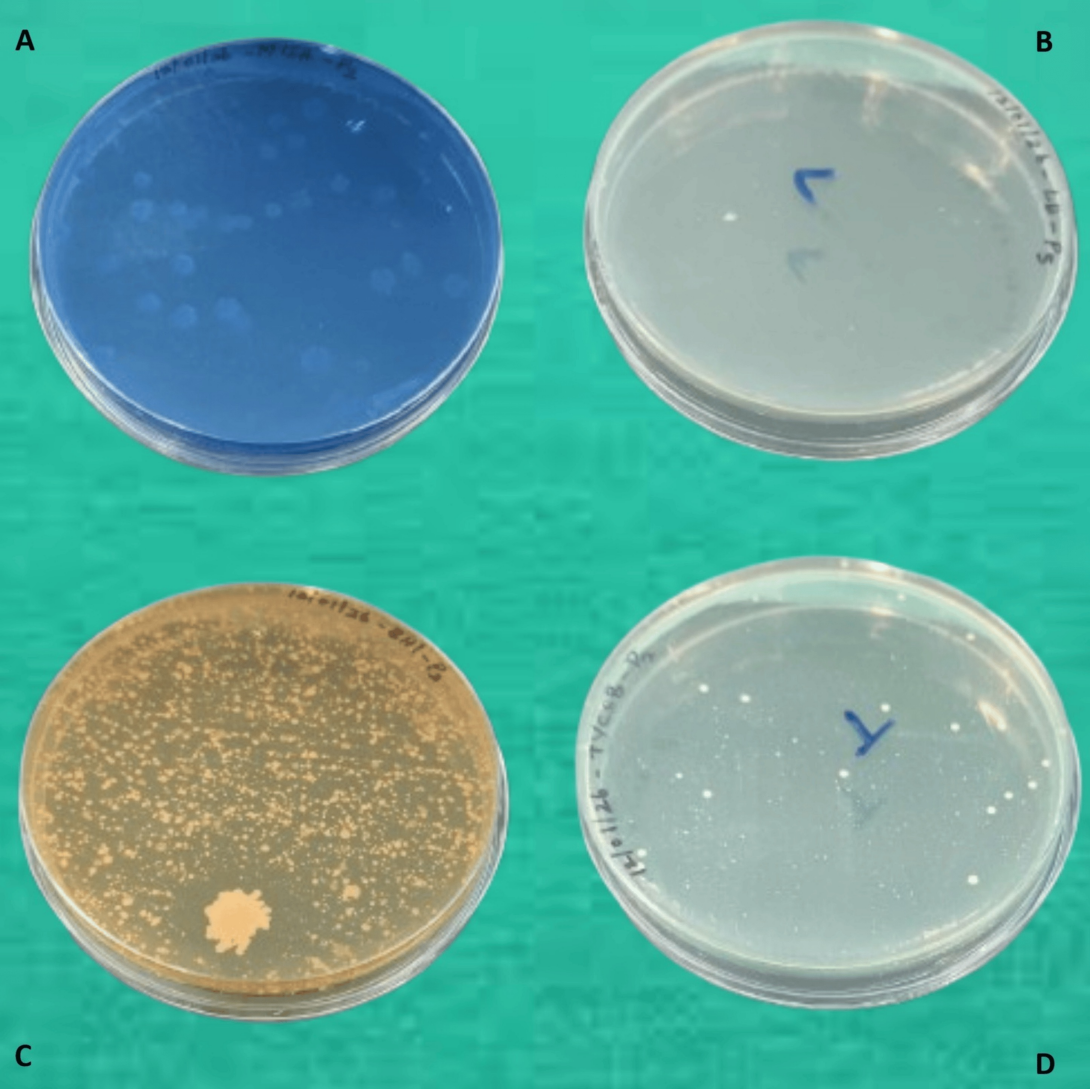
Culture plates showing variable bacterial presence on selective media at baseline (A) *Streptococcus salivarius*. (B) *Lactobacillus sporogenes*. (C) *Streptococcus oralis*. (D) *Streptococcus mutans*

**Figure 2:**
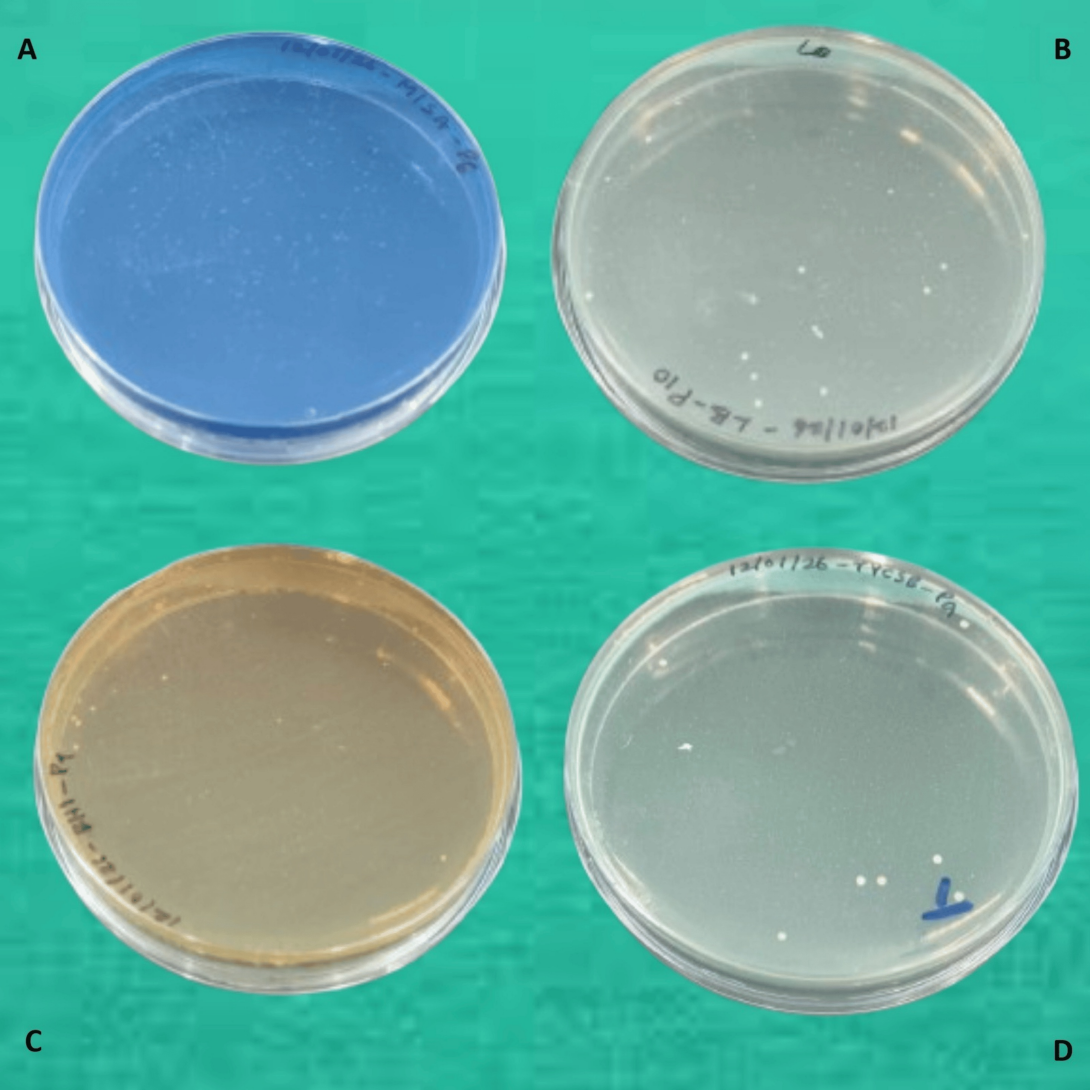
Culture plates showing variable bacterial presence on selective media after 240 days of Hetafu mouthwash use (A) *Streptococcus salivarius*. (B) *Lactobacillus sporogenes*. (C) *Streptococcus oralis*. (D) *Streptococcus mutans*

In the microbiology laboratory, each plaque sample was diluted to obtain a uniform suspension. The diluted samples were then inoculated onto selective culture media specific for the target microorganisms. *Streptococcus mutans* was cultured on Tryptone Yeast Extract Cystine Sucrose Bacitracin (TYCSB) agar under anaerobic conditions for 24-48 hours. *Streptococcus salivarius* was grown on Mitis Salivarius agar, while *Lactobacillus* species were cultured on Lactobacillus Selective agar, both incubated at 37°C for 24-48 hours. *Streptococcus oralis* was cultured on Brain Heart Infusion agar supplemented with colistin (10 µg/mL) and oxolinic acid (5 µg/mL), incubated at 37°C for 24-48 hours. Following incubation, colony‑forming units were quantified for each organism (Figures 1, 2).

These culture results, obtained from every swab collected at each time interval, were subsequently tabulated and used for comparative analysis across the study period.

Statistical analysis was performed using SPSS software version 26.0 (IBM Corp., Armonk, NY). Changes in bacterial counts over time (baseline, 60 days, and 240 days) were analyzed using the Friedman test. When significant differences were observed, post hoc pairwise comparisons were performed using Dunn’s test with Bonferroni modification. A p-value of less than 0.05 was considered statistically significant.

## Results

The results of the study at the three follow-up assessments, each comprising 20 participants, are as follows: *Streptococcus mutans* counts demonstrated a significant and progressive reduction over time. The mean values decreased from 186.00 at the first follow-up to 133.35 at the second follow-up, with a marked decline to 19.30 by the third follow-up (Table 1). Median values followed a similar downward trend, indicating consistency in the reduction across participants. The overall comparison revealed a statistically significant difference in *Streptococcus mutans* counts across the three follow-up periods (p = 0.001), confirming a significant reduction over successive follow-ups.

**Table 1:** Comparison of colony-forming unit counts of Streptococcus mutans in dental plaque across three follow-up assessments using Friedman’s test The P-value represents the comparison of median values across the three time points

	Number of participants	Mean	Standard deviation	Median	Minimum	Maximum	Test statistics	P-value
Baseline-firstday	20	186.0000	133.82628	275.0000	10.00	300.00	39.519	0.001
60th day	20	133.3500	103.81019	145.0000	7.00	260.00		
240th day	20	19.3000	23.76220	8.0000	0.00	60.00		

Pairwise comparisons with Bonferroni adjustment revealed statistically significant differences in *Streptococcus mutans* counts between all follow-up periods. The third follow-up showed significantly lower *Streptococcus mutans* levels compared with both the second follow-up (adjusted p = 0.004) and the first follow-up (adjusted p < 0.001), indicating a substantial reduction over time. Additionally, a significant difference was observed between the second and first follow-ups (adjusted p = 0.008) (Table 2).

**Table 2:** Post hoc pairwise comparisons of Streptococcus mutans colony-forming unit counts across follow-up time points using Dunn’s test with Bonferroni modification

Sample 1	Sample 2	Mean rank difference	Standard error	P-value	Adjusted p-value
240th day	60th day	1.025	0.316	0.001	0.004
240th day	Baseline-first day	1.975	0.316	<0.001	<0.001
60th day	Baseline-first day	0.950	0.316	0.003	0.008

At the three follow-up assessments, each including 20 participants, *Lactobacillus sporogenes* counts showed a modest increase over time. The mean values were 98.40 at the first follow-up and remained comparable at the second follow-up (99.10), followed by an increase to 109.35 at the third follow-up. Median values also demonstrated a gradual rise from 40.00 at the first follow-up to 42.50 at the second follow-up and 50.00 at the third follow-up. The overall comparison across the three follow-up periods was statistically significant (p = 0.001), indicating a significant change in *Lactobacillus* species counts over successive follow-ups (Table 3).

**Table 3:** Comparison of colony-forming unit counts of Lactobacillus sporogenes in dental plaque across three follow-up assessments using Friedman’s test The p-value represents the comparison of median values across the three time points

	Number of participants	Mean	Standard deviation	Median	Minimum	Maximum	Test statistics	P-value
Baseline-firstday	20	98.4000	112.31647	40.0000	3.00	300.00	19.937	0.001
60th day	20	99.1000	102.61471	42.5000	5.00	300.00		
240th day	20	109.3500	111.23483	50.0000	10.00	300.00		

Bonferroni-adjusted pairwise comparisons of *Lactobacillus sporogenes* counts across the three follow-up assessments demonstrated a significant increase only between the first and third follow-ups. The third follow-up showed significantly higher *Lactobacillus* counts compared with the first follow-up (adjusted p < 0.001). In contrast, no statistically significant differences were observed between the first and second follow-ups (adjusted p = 0.246) or between the second and third follow-ups after correction for multiple comparisons (adjusted p = 0.081) (Table 4).

**Table 4:** Post hoc pairwise comparisons of Lactobacillus sporogenes colony-forming unit counts across follow-up time points using Dunn’s test with Bonferroni modification

Sample 1	Sample 2	Mean rank difference	Standard error	P-value	Adjusted p-value
240th day	60th day	-0.550	0.316	0.082	0.246
240th day	Baseline-first day	-1.250	0.316	0.000	0.000
60th day	Baseline-first day	-0.700	0.316	0.027	0.081

Across the three follow-up assessments (n = 20 per follow-up), *Streptococcus oralis* counts demonstrated a pronounced and progressive decline over time. The mean count decreased from 243.50 at the first follow-up to 142.50 at the second follow-up and further to 28.00 at the third follow-up. Median values reflected the same downward trend, indicating consistency across participants. The overall comparison across the three follow-up periods was statistically significant (p = 0.001), confirming a significant reduction in *Streptococcus oralis* levels over successive follow-ups (Table 5).

**Table 5:** Comparison of colony-forming unit counts of Streptococcus oralis in dental plaque across three follow-up assessments using Friedman’s test The p-value represents the comparison of median values across the three time points

	Number of participants	Mean	Standard deviation	Median	Minimum	Maximum	Test statistics	P-value
Baseline-firstday	20	243.5000	84.80783	300.0000	30.00	300.00	39.519	0.001
60th day	20	142.5000	67.73594	100.0000	30.00	250.00		
240th day	20	28.0000	22.38420	25.0000	0.00	70.00		

Bonferroni-adjusted pairwise comparisons revealed statistically significant differences in *Streptococcus oralis* counts between all follow-up periods. The third follow-up demonstrated significantly lower *Streptococcus oralis* levels compared with both the second follow-up (adjusted p = 0.004) and the first follow-up (adjusted p < 0.001). In addition, a significant difference was observed between the second and first follow-ups (adjusted p = 0.008) (Table 6).

**Table 6:** Post hoc pairwise comparisons of Streptococcus oralis colony-forming unit counts across follow-up time points using Dunn’s test with Bonferroni modification

Sample 1	Sample 2	Mean rank difference	Standard error	P-value	Adjusted p-value
240th day	60th day	1.025	0.316	0.001	0.004
240th day	Baseline-first day	1.975	0.316	0.000	0.000
60th day	Baseline-first day	0.950	0.316	0.003	0.008

Across the three follow-up assessments (n = 20 per follow-up), *Streptococcus salivarius* counts demonstrated a progressive increase over time. The mean values rose from 167.00 at the first follow-up to 182.75 at the second follow-up and further to 209.25 at the third follow-up. Median values showed a similar upward trend, indicating a consistent increase across participants. The overall comparison across the three follow-up periods was statistically significant (p = 0.001), indicating a significant increase in *Streptococcus salivarius* counts over successive follow-ups (Table 7).

**Table 7:** Comparison of colony-forming unit counts of Streptococcus salivarius in dental plaque across three follow-up assessments using Friedman’s test The p-value represents the comparison of median values across the three time points

	Number of participants	Mean	Standard deviation	Median	Minimum	Maximum	Test statistics	P-value
Baseline-firstday	20	167.0000	107.02189	200.0000	20.00	300.00	24.862	0.001
60th day	20	182.7500	103.65016	205.0000	45.00	300.00		
240th day	20	209.2500	103.01092	275.0000	55.00	300.00		

Bonferroni-adjusted pairwise comparisons of *Streptococcus salivarius* counts revealed significant differences primarily involving the third follow-up assessment. The third follow-up showed significantly higher *Streptococcus salivarius* counts compared with both the first follow-up (adjusted p < 0.001) and the second follow-up (adjusted p = 0.022). In contrast, no statistically significant difference was observed between the first and second follow-ups after adjustment for multiple comparisons (adjusted p = 0.399) (Table 8).

**Table 8:** Post hoc pairwise comparisons of Streptococcus salivarius colony-forming unit counts across follow-up time points using Dunn’s test with Bonferroni modification

Sample 1	Sample 2	Mean rank difference	Standard error	P-value	Adjusted p-value
240th day	60th day	-0.475	0.316	0.133	0.399
240th day	Baseline-first day	-1.325	0.316	0.000	0.000
60th day	Baseline-first day	-0.850	0.316	0.007	0.022

## Discussion

Orthodontic patients face unique challenges in maintaining microbial balance due to the plaque‑retentive nature of fixed appliances [2]. This pilot study set out to determine whether Hetafu mouthwash, formulated with probiotics, prebiotics, essential oils, and enamel-supporting minerals, could influence the growth patterns of key beneficial and cariogenic bacteria during treatment.

Previous investigation by Lakkoju et al. in 2024 demonstrated that Hetafu candy consumption, which is formulated with probiotics, significantly reduced *Streptococcus mutans* and Actinomycetes levels over an eight‑week period, indicating a favorable shift toward a healthier oral microbiome [5].

Also, a multi‑cluster clinical study conducted by the same author in 2025 on Hetafu “Pink Smart Gummies” reported improvements in several dental health parameters, which include salivary flow, halitosis, gingival inflammation, plaque accumulation, and caries activity. These gummies, enriched with probiotics (*Bacillus coagulans*, *Lacticaseibacillus rhamnosus*, and *Lactiplantibacillus plantarum*), prebiotics (FOS), sugar alcohols (xylitol, maltitol, and sorbitol), and essential oils, demonstrated significant clinical benefits compared to controls [6].

Findings by Albardawel et al. further support the concept of microbiome‑modulating adjuncts during orthodontic treatment, as their randomized controlled trial showed that probiotic supplementation significantly reduced plaque accumulation, gingival inflammation, and *Streptococcus mutans* levels in adults wearing fixed appliances [11]. A systematic review and meta‑analysis by Chen et al. (2023) synthesized evidence on the use of probiotics in orthodontic patients and concluded that probiotic supplementation can produce measurable oral health benefits during fixed appliance therapy. Their analysis showed consistent reductions in plaque accumulation, gingival inflammation, and *Streptococcus mutans* levels across multiple clinical trials. Although the magnitude of improvement was varying and it depended on probiotic strain and study design, the overall findings supported the role of probiotics in regulating the oral microenvironment and improving periodontal parameters in orthodontic populations [12].

No previous study has evaluated the effect of Hetafu mouthwash on the oral microbiota of orthodontic patients. This pilot trial is the first to examine how Hetafu mouthwash influences both beneficial commensals and cariogenic bacteria in a population uniquely prone to biofilm retention. The findings demonstrate that the mouthwash produced measurable shifts in the oral microbial profile, suggesting a modulatory influence on biofilm composition during fixed appliance therapy.

A significant and progressive reduction in *Streptococcus mutans* was observed across all time points. This decline aligns with previous investigations on Hetafu formulations, which reported reductions in cariogenic species following the routine consumption of functional gummies enriched with probiotics, essential oils, and sugar alcohols [5]. The antimicrobial properties of essential oils and probiotic-derived metabolites may collectively inhibit *Streptococcus mutans* adhesion and acidogenicity, thereby contributing to the marked reduction seen in this study. Similar trends have been documented in earlier work demonstrating that probiotic and bioactive supplements can suppress pathogenic streptococci and shift the oral microenvironment toward a less cariogenic state [13].

*Lactobacillus sporogenes* showed a slight but statistically significant increase between the start of the study and the final follow-up. The rise observed here may be advantageous, as *Lactobacillus sporogenes* functions as a probiotic that helps sustain microbial equilibrium in the oral cavity [14]. During orthodontic treatment, where brackets and wires encourage biofilm accumulation, the growth of such beneficial bacteria could support a healthier microbial community rather than fostering pathogenic species. To clarify the clinical relevance of this finding, further strain-specific analysis would be useful.

Among the beneficial commensals, *Streptococcus salivarius* exhibited a steady and significant increase over the study period. This finding is consistent with the known probiotic‑supportive effects of functional supplements, which may enhance the colonization of health‑associated species [5]. *Streptococcus salivarius* plays a key role in maintaining oral ecological stability, producing bacteriocins that inhibit cariogenic organisms and supporting mucosal health [15]. Its progressive rise suggests that Hetafu mouthwash may promote a more resilient commensal community, counterbalancing the reduction in pathogenic bacteria.

*Streptococcus oralis* showed a marked reduction at all evaluated time points following the use of mouthwash. This decline is likely linked to the antimicrobial properties of the rinse, which can selectively suppress bacteria that are more vulnerable within the oral biofilm. The decrease in *Streptococcus oralis* may also indicate broader ecological shifts in microbial composition, where changes in local conditions lead to the suppression of certain species. Previous research has demonstrated that antimicrobial agents can reshape biofilm communities, often resulting in reductions of specific bacterial groups [16]. Further longitudinal studies are needed to assess whether this decline persists and to clarify its overall influence on the oral microbial environment.

Overall, the microbial trends observed in this study suggest that Hetafu mouthwash exerts a modulatory rather than purely antimicrobial effect, reducing key cariogenic species while supporting the growth of *Streptococcus salivarius*, a beneficial commensal. This dual action is particularly relevant to orthodontic patients, who face a heightened risk of plaque deposition, enamel demineralization, and gingival inflammation due to appliance‑induced retention sites. While professional interventions such as Guided Biofilm Therapy (GBT) provide episodic plaque control, daily nutraceutical supplements may offer continuous microbial support between dental visits [17,18].

The limitations of this study include a small sample, which is consistent with the exploratory nature of a pilot study. The study did not have a control group, and so, it is not possible to differentiate the effects of the mouthwash from natural microbial fluctuations during orthodontic treatment. Additionally, dietary habits and oral hygiene compliance, though controlled through instructions, may have introduced variability.

Future research should incorporate larger randomized controlled trials, include molecular microbiome profiling, and evaluate clinical outcomes such as plaque index, gingival inflammation, and white spot lesion development. Assessing the long‑term stability of microbial changes after the discontinuation of the mouthwash would also provide valuable insight into their sustained impact.

## Conclusions

The daily use of Hetafu mouthwash for 240 days produced a measurable modulation of the oral microbial profile in orthodontic patients. The mouthwash was associated with a substantial reduction in *Streptococcus mutans* and a marked increase in *Streptococcus salivarius*, indicating a shift toward a less cariogenic and more commensal‑supportive biofilm environment. A consistent reduction in *Streptococcus oralis* levels was observed, along with a modest increase in *Lactobacillus sporogenes*, which may be considered beneficial in promoting microbial balance. The overall microbial pattern suggests that the Hetafu mouthwash exerts a regulatory rather than purely antimicrobial effect.

As a pilot study, the results provide preliminary evidence supporting the microbial benefits of Hetafu mouthwash; however, larger randomized controlled trials with molecular microbiome analysis are needed to confirm these outcomes and further clarify their clinical significance.
